# An observation-based perspective of winter haze days in four major polluted regions of China

**DOI:** 10.1093/nsr/nwy118

**Published:** 2018-10-17

**Authors:** Lu Mao, Run Liu, Wenhui Liao, Xuemei Wang, Min Shao, Shaw Chen Liu, Yuanhang Zhang

**Affiliations:** 1 State Key Joint Laboratory of Environmental Simulation and Pollution Control, Beijing Innovation Center for Engineering Science and Advanced Technology, College of Environmental Sciences and Engineering, Peking University, Beijing 100871, China; 2 Institute for Environmental and Climate Research, Jinan University, Guangzhou 510632, China; 3 Guangdong University of Finance, Guangzhou 510521, China

**Keywords:** haze, visibility, global warming

## Abstract

An observation-based approach is used to examine key characteristics of winter haze days in four major polluted regions in China. Major findings in this study are: first, there was no significant trend in the number of winter haze days in most provinces and districts in eastern China from 1973 to 2012, contrary to the 2.5-fold increase in the emissions of particulate matter and its precursors (PM emissions) in the same period of time. Second, meteorological and climate conditions rather than PM emissions are in control of the interannual variabilities and trends of winter haze days. These interannual variabilities (ranging from 24 to 125%) pose a substantial masking effect that must be overcome by any control of PM emissions before its impact becomes statistically detectable. Finally, we find that global warming may have contributed significantly to the trend of winter haze days in eastern China.

## INTRODUCTION

Haze has been a serious environmental problem in China over the last few decades. These fine particles contain toxic substances that affect respiratory and circulatory systems, with detrimental impacts on human health [1–3]. Haze also has a degradation effect on visibility, sometimes serious enough to become air and ground traffic hazards [4–6]. The majority of haze days in four major polluted regions of China (Supplementary Fig. 1, available as Supplementary Data at *NSR* online), namely Beijing-Tianjin-Hebei (BTH), Yangtze River Delta (YRD), Pearl River Delta (PRD) and Sichuan Basin (SCB), occur in the winter months of November through February, with monthly differences exceeding an order of magnitude (Supplementary Fig. 2, available as Supplementary Data at *NSR* online). It is well known that the fundamentals of accumulation of air pollutants are their emissions, transport (atmospheric dynamics), transformation (chemical and physical) and deposition. In question are the specific mechanisms and their quantitative contributions to the accumulation of air pollutants. Since little abrupt monthly changes in deposition, transformation or emission of haze were expected to occur, stagnant meteorological conditions with shallow surface inversions conducive to the accumulation of haze, which were known to have frequent and large variability in winter months, were shown in many studies to be the major factors contributing to high winter haze days [6–14].

A number of recent studies have shown that global warming is having a significant adverse effect on the haze problem in China [11,13,15–18]. This notion is not surprising, as a weakening of seasonal and winter mean surface winds has been reported in many regions over land in last few decades during which global temperature has increased significantly [19]. In addition, a robust enhancement in vertical stability with global warming has been found in observations as well as results of climate models [20].

Specifically, Wang *et al.* [11] hypothesized that decreasing Arctic sea ice (ASI) could be an important contributor to the recent increased haze days in eastern China, and about 45–67% of the interannual to interdecadal variability of winter haze days could be explained by ASI variability. The reduction of ASI favors less cyclone activity and more stable atmosphere conducive to haze buildup [21]. Other studies suggested that high winter haze days are associated with high El Niño-Southern Oscillation (ENSO) indexes [22], low East Asian Winter Monsoon (EAWM) indexes [12], high Pacific Decadal Oscillation (PDO) indexes [23] and high Arctic Oscillation (AO) indexes [13]. In addition, the increasing number of haze days in North China may be also associated with the weakening trend of surface wind [24,25], high relative humidity [26], low sea-level pressure [10] and increasing trend in surface temperature [10]. Finally, Cai *et al.* [13] projected a 50% increase in the frequency of extreme haze events when comparing historical (1950–99) to future (2050–99) climate under Representative Concentration Pathway 8.5. Remarkably, all global warming effects cited above tend to worsen the haze problem.

The studies cited above indicate that meteorological and climate conditions contribute prominently to the interannual variability of haze days. In this study, an observation-based approach is utilized to evaluate the effectiveness of emission control in reducing the number of winter haze days in the four major polluted regions of China. We will identify and try to quantify major mechanisms/processes in control of the interannual variability of winter haze days by examining the key characteristics of winter haze days and their relationship to emissions of air pollutants, and to meteorological and climate conditions.

Haze days in this study are derived from observations of visibility [27]. Deriving haze days from visibility has an important advantage because observations of visibility are available over a long period of time compared to observations of PM_2.5_ or PM_10_, of which reliable measurements in China are too few for any meaningful trend analysis. However, it should be noted that haze days derived this way do not have a simple proportional relationship with PM concentrations, because the optical properties of PM depend strongly on many environmental factors, such as relative humidity and aerosol composition. Therefore, caution must be taken that conclusions drawn from the analysis of haze days do not necessarily apply to aerosols or PM, particularly for quantitative conclusions.

## CHARACTERISTICS OF WINTER HAZE DAYS

**Figure 1.**
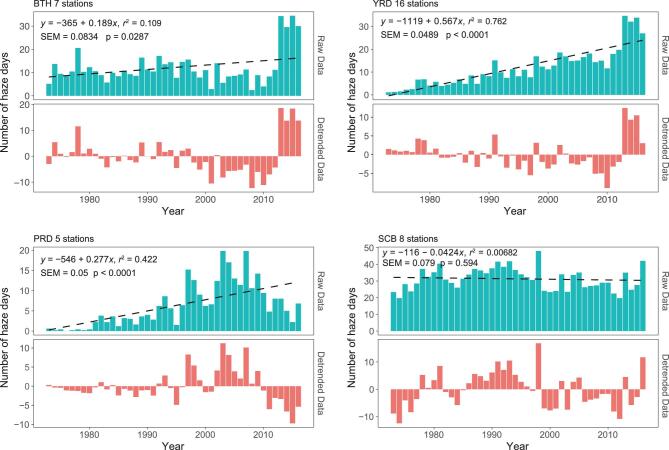
Number of winter haze days (November–December–January–February) in four major polluted regions of China during the period 1973–2016. Absolute values are shown in blue, detrended values in red. Dashed lines denote linear regressions, and equations are statistics for the regressions.

Figure 1 shows time series of the winter haze days (defined as visibility <5 km and relative humidity <95% [27]) in BTH, YRD, PRD and SCB during the winter months of November, December, January and February (NDJF) (ND and JF from consecutive years) during the period 1973–2016. NDJF is chosen because the majority (64%) of haze days in BTH occur in this period. For ease of comparison with one another, the same period is chosen for other three regions, although, in certain areas like YRD, the majority is about 57%.

At first glance, the time series of winter haze days of four regions look quite different from one another. BTH, YRD and PRD have significant but different values of linear trends, while no significant trend exists in SCB. The trend in YRD looks closest to what is expected, namely with a quasi-linear trend of 4.7%/year that resembles a typical trend of PM emissions (Supplementary Fig. 3, available as Supplementary Data at *NSR* online). However, only PRD shows a significant reduction in winter haze days in the last decade, which appears to be consistent with the reduction of PM_2.5_ concentration reported extensively over urban areas in China [28].

A surprising phenomenon can be seen in BTH: winter haze days remained essentially constant at a moderate value of 10 days (i.e. no trend) during a 40-year span from 1973 to 2012. In the meantime, PM emissions in BTH increased by about 2.5-fold (Supplementary Fig. 3, available as Supplementary Data at *NSR* online). This stark discrepancy between trends of haze days and PM emissions strongly suggests that factors/processes other than PM emissions are in control of the trend as well as interannual variability of winter haze days in the 40-year span. What are these factors/processes? As mentioned in the introduction, concentrations of air pollutants are primarily determined by emissions, transport, transformation and deposition, of which only atmospheric transport such as stagnant meteorological conditions exhibit large variability at wide ranges of temporal and spatial scales. Therefore, an obvious candidate for the factors/processes is that certain meteorological and climate conditions such as the mixing height of the mixed layer, surface layer wind and/or relative humidity were in control of the interannual variability of winter haze days during the 40-year span. The control was so overwhelming that it completely masked the expected increase in the winter haze days due to the 2.5-fold increase in PM emissions from 1973 to 2012. This has a surprising beneficial ramification: during the 40-year span, residents in BTH apparently did not suffer the expected detrimental effects of increased PM emissions, thanks to the particular meteorological and climate conditions. Nonetheless, the full impact of increased PM emissions appeared to have returned to BTH after 2012, as shown in the conspicuous 4-year surge of haze days in 2013–16.

Which meteorological and climate parameters control the genesis of haze days and how? This question can be addressed by comparing key meteorological and climate parameters of haze days to those of clean days, as illustrated in Table 1. In this comparison, we select those years with approximately equal numbers of haze days and clean days (within 20% of each other) such that the meteorological parameters can be isolated from the climate parameters and PM emissions, as the latter two are in the same years and thus identical. Table 1 shows that meteorological parameters of haze days relative to those of clean days are characterized by low wind, high humidity, low atmospheric pressure, high atmospheric stability, shallow boundary layer, etc. These characteristics are highly similar in four regions, particularly for atmospheric pressure, temperature and winds. These results are consistent with the general understanding of mechanisms and processes associated with the accumulation of air pollutants. Furthermore, results in Table 1 are in excellent agreement with previous findings [7–10,13,14,26].

**Table 1.** Differences in key meteorological parameters between haze days and clean days.

		Polluted days		Clean days			
Region	Meteorological parameter	Mean	Std	Mean	Std	Difference	*P*-value (bootstrap)
**BTH**	**RH** ^a^	76.5	8.1	56.5	10.2	20.0	<0.001
	**SLP** ^b^	102 429.7	513.9	103 181.1	501.1	–751.4	<0.001
	**T_2m** ^c^	269.9	4.5	263.3	5.8	6.6	<0.001
	**ws_10m** ^d^	2.7	1.1	4.4	1.4	–1.7	<0.001
	**u_10m** ^e^	1.1	1.4	2.8	1.4	–1.7	<0.001
	**v_10m** ^f^	–0.4	1.9	–2.7	1.9	2.3	<0.001
	**delT_925_1000** ^g^	–1.9	1.2	–3.1	0.5	1.1	<0.001
	**BLH** ^h^	221.4	50.9	562.8	191.8	–341.4	<0.001
	**hgt_500** ^i^	5561.3	74.2	5469.5	90.8	91.8	<0.001
	**omega_850** ^j^	4.1E-2	5.7E-2	18.8E-2	7.1E-2	−14.7E-2	<0.001
**YRD**	**RH**	75.5	9.5	62.5	11.0	13.0	<0.001
	**SLP**	102 261.1	410.0	102 948.5	475.6	–687.4	<0.001
	**T_2m**	281.8	3.8	276.4	5.8	5.4	<0.001
	**ws_10m**	2.6	0.9	3.7	1.1	–1.1	<0.001
	**u_10m**	–0.4	1.3	0.4	1.7	–0.8	0.0014
	**v_10m**	–0.4	1.8	–1.8	2.1	1.4	<0.001
	**delT_925_1000**	–2.1	0.9	–2.9	1.0	0.8	<0.001
	**BLH**	316.7	73.5	498.3	148.6	–181.6	<0.001
	**hgt_500**	5689.7	52.0	5665.4	76.8	24.4	0.0285
	**omega_850**	2.1E-2	3.9E-2	7.6E-2	4.7E-2	−5.6E-2	<0.001
**PRD**	**RH**	75.5	12.5	62.2	13.3	13.2	<0.001
	**SLP**	101 762.2	272.0	102 388.3	363.3	–626.1	<0.001
	**T_2m**	290.1	2.6	285.3	4.0	4.8	<0.001
	**ws_10m**	2.6	0.8	4.6	1.1	–2.0	<0.001
	**u_10m**	–1.5	0.6	–2.3	0.6	0.8	<0.001
	**v_10m**	–0.7	1.7	–3.7	1.6	3.0	<0.001
	**delT_925_1000**	–3.3	0.6	–3.0	0.8	–0.3	0.0402
	**BLH**	356.7	69.1	526.7	144.1	–170.0	<0.001
	**hgt_850**	1529.2	19.0	1554.3	14.7	–25.2	<0.001
	**omega_850**	−2.3E-2	4.8E-2	5.7E-2	4.6E-2	−8.0E-2	<0.001
**SCB**	**RH**	67.1	9.8	69	12.2	–1.96	0.1747
	**SLP**	102 301.3	583.5	102 813.0	572.9	–511.7	<0.001
	**T_2m**	277.8	3.3	275.1	4.3	2.6	<0.001
	**ws_10m**	2.3	0.7	2.2	0.8	0.1	0.1825
	**u_10m**	–1.1	1.3	–1.2	1.2	0.1	0.6488
	**v_10m**	–0.1	1.0	–0.1	1.0	0.0	0.8998
	**delT_925_1000**	–3.8	0.3	–3.9	0.3	0.2	<0.001
	**BLH**	304.1	61.0	406.0	122.3	–101.8	<0.001
	**hgt_850**	1526.0	40.3	1556.3	37.9	–30.3	<0.001
	**omega_500**	2.8E-2	9.3E-2	6.3E-2	8.7E-2	−3.5E-2	0.0044

^a^Relative humidity, in unit of %.

^b^Sea-level pressure, in unit of Pa.

^c^Temperature at 2 m, in unit of K.

^d^Wind speed at 10 m, in unit of m/s.

^e^Zonal wind speed at 10 m, in unit of m/s.

^f^Meridional wind speed at 10 m, in unit of m/s.

^g^Temperature difference between 925 and 1000 hPa (T@925–T@1000), in unit of K.

^h^Boundary layer height, in unit of m.

^i^Geopotential height at 500 hPa, in unit of m.

^j^Vertical velocity at 850 hPa, in unit of Pa/s.

The lack of any significant trend in haze days for a long period of time is not limited to BTH. Similar phenomena are present in the majority number of provinces and districts in eastern China, particularly in Heilongjiang, Liaoning, Shandong, Shanxi, Hubei and Hunan provinces as well as SCB (Fig. 1 and Supplementary Fig. 4, available as Supplementary Data at *NSR* online). This phenomenon is also highly robust, as evident by the fact that it remains the same in a number of cases for which different criteria are used to classify winter haze days, including choosing 10-km visibility as the threshold for the haze days, a 90% relative humidity threshold, weighing the haze days by the value of visibility, etc. Moreover, the lack of any significant trend also can be seen in previous studies; for instance, in the study by Wang *et al.* [11], we notice that time series of winter haze days in entire eastern China showed no significant trend over a 37-year span between 1979 and 2005, agreeing well with this study.

A close inspection of the detrended data reveals that the lack of trend in BTH and other regions can be attributed to a depression of the winter haze days during the period 1999–2012. The depression apparently diminishes gradually toward southeastern China, as shown in YRD and PRD (Fig. 1). Nevertheless, the depression remains visible in the detrended data, particularly for two deeper parts of the depression: 1999–2001 and 2008–12, which are visible in all four regions (Fig. 1 and Supplementary Fig. 4, available as Supplementary Data at *NSR* online). Since the annual PM emissions during the 1999–2012 depression are significantly higher than those during 1973–98 (Supplementary Fig. 3, available as Supplementary Data at *NSR* online), the persistence of the depression again provides strong evidence supporting the notion that meteorological and climate conditions rather than PM emissions are in control of the interannual variability of winter haze days; and that the control is at least synoptic in scale. Interestingly, in PRD, the part of depression before 2005 has mostly diminished, while the part of depression near 2008–12 remains robust. Furthermore, the 2008–12 depression seems to have extended all the way to 2016 in PRD. In this regard, since the 2008–12 depression was caused by certain meteorological and climate conditions, it is tantalizing to propose that the extension to 2016 was also caused, at least partially, by these conditions. This proposal, if confirmed, would have a profound implication for the control strategies of haze days in PRD. In this context, we notice that a majority of coastal stations, in addition to those in PRD, exhibit similar post-2007 improvements in the haze problem, such as Jinzhou, Tsingtao, Shanghai and Hangzhou (Supplementary Fig. 5, available as Supplementary Data at *NSR* online). This suggests that changes of large-scale land–ocean circulations in the last decade could be an important contributor to the post-2007 improvement.

What climate conditions caused the depression of winter haze days during 1999–2012 (named hereafter as Period 2)? How different are these conditions compared to those of non-depression periods, namely 1973–98 (named as Period 1) and 2013–16 (named as Period 3)? These two questions can be addressed by comparing the climate conditions during Period 2 to those of Periods 1 and 3. As the depression represents the period least conducive to the genesis of haze days, according to previous studies [12,13,21–23], one would expect its PDO, AO, ENSO and GT (global temperature) to have the lowest values among the three periods, while its EAWM and ASI have the highest values. Remarkably, this is indeed the case. An example is presented in Table 2, which shows the difference between Period 2 and Period 3. These results are further substantiated by elevated significant levels for all six climate parameters (Table 3) when Period 2 is replaced by the two deeper parts of the depression: 1999–2000 and 2009–11. As a brief summary for this paragraph, our analysis shows that the depression of winter haze days during 1999–2012 is caused by a combination of relative small (relative to non-depression periods) values of PDO, AO, ENSO and GT, in addition to relative large values of EAWM and ASI.

**Table 2.** Differences in key climate parameters between Period 2 and Period 3.

	Period 2		Period 3			
Climate						*P*-value
Parameter	Mean	Std	Mean	Std	Difference	(bootstrap)
**PDO**	–0.49	1.03	1.02	0.96	–1.51	<0.001
**AO**	–0.18	1.3	0.28	1.29	–0.47	0.20628
**ENSO**	–0.22	0.92	0.43	1.17	–0.65	0.02532
**GT**	–0.19	0.95	0.49	0.98	–0.68	0.01592
**EAWM**	1.62	1.38	1.19	1.21	0.43	0.26268
**ASI**	–0.01	0.98	–0.34	1.48	0.32	0.29968

**Table 3.** Same as Table 2, but Period 2 is replaced by 1999–2000 and 2009–11 (Period 2*).

	Period 2*		Period 3			
Climate						*P*-value
Parameter	Mean	Std	Mean	Std	Difference	(bootstrap)
**PDO**	–0.93	0.98	1.02	0.96	–1.96	<0.001
**AO**	–0.48	1.56	0.28	1.29	–0.76	0.11844
**ENSO**	–0.66	1.01	0.43	1.17	–1.09	0.00672
**GT**	–0.75	0.76	0.49	0.98	–1.25	<0.001
**EAWM**	1.8	1.54	1.19	1.21	0.62	0.18992
**ASI**	0.41	0.74	–0.34	1.48	0.75	0.05668

## TRENDS OF WINTER HAZE DAYS

As trends of PM emissions tend to be quasi-linear (Supplementary Fig. 3, available as Supplementary Data at *NSR* online), linear trends of winter haze days are very useful for comparison with PM emissions. The linear trends of winter haze days in BTH, YRD, PRD and SCB shown in Fig. 1 are 1.5%/year, 4.7%/year, 4.4%/year and no significant trend, respectively. In comparison, corresponding trends of PM emissions in the four respective regions (Supplementary Fig. 3, available as Supplementary Data at *NSR* online) are 2.1%/year, 2.4%/year, 2.65%/year and 1.7%/year. None of the trends of haze days in four regions compares well with their corresponding trends of PM emissions, especially when the large interannual variability of haze days is taken into account. This again underscores the notion that climate and meteorological conditions rather than the PM emissions play the predominant role in controlling the interannual variability and thus the trends of haze days.

Li *et al.* [29] found that linear trends of aerosol optical depth (AOD) derived from observations of sunshine hours, solar radiation and visibility in eastern China were about (0.95∼1.4)%/year during the period 1973–2005. This trend is less than half of average trend of winter haze days (about 2.5%/year) in the three eastern regions, namely BTH, YRD and PRD, for the same period. Why is there such a large difference between the trends of AOD and winter haze days? Haze days are closely related to AOD in the surface mixed layer. However, AOD, being a vertical integral of aerosol extinction (scattering and absorption), unlike haze days, is not sensitive to changes in the height and stability of the mixed layer. It follows that the difference between the trends of haze days and AOD can be regarded as a measure of the impact on the surface concentration of aerosols due to changes in the height and stability of the mixed layer. This is consistent with the significant trends in the lapse rate and vertical velocity (Supplementary Fig. 6, available as Supplementary Data at *NSR* online), which are most likely caused by global warming [20].

In summary of this section, long-term changes in the lapse rate and vertical velocity, which are most likely driven by global warming, may have contributed significantly to trend of winter haze days in eastern China.

## INTERANNUAL VARIABILITY OF WINTER HAZE DAYS

**Figure 2.**
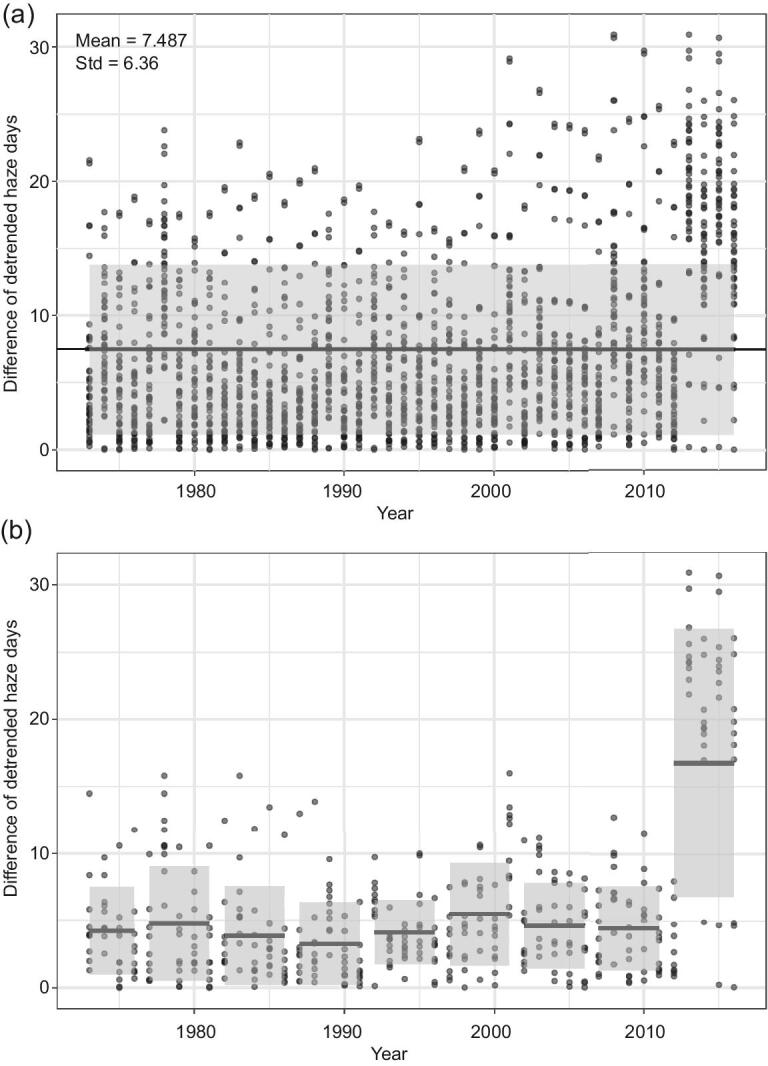
Interannual variability of winter haze days in Beijing-Tianjin-Hebei (BTH) for 44-year span (a) and 10-year span (b) during the period 1973–2016. Solid bars denote mean values, shaded areas are 1-standard deviations.

Statistically, the interannual variability of detrended data of haze days can be defined for various intervals of time, such as 10 consecutive years and the entire 44-year period. The latter is the conventional definition; the former is also useful, as shown in the following. Since the interannual variability is controlled by meteorological and climate conditions, which we lack the capability to predict at this point in time, the interannual variability of a specific period poses as a masking effect (noise) that must be overcome by any change in PM emissions within that period before its impact (signal) becomes statistically detectable. Taking BTH as an example, the interannual variability of the entire 44 years can be evaluated by taking the difference between an individual year and the remaining 43 years (Fig. 2a); and the interannual variability of 10 consecutive years can be evaluated by taking the difference between an individual year and 9 earlier years (Fig. 2b). All evaluations are straightforward except for years earlier than 1983 of the 10-year case, for which differences with preceding (*n*) years and following (9 – *n*) years are taken.

For BTH, the mean value of 44-year interannual variability is about 7.5 haze days (Fig. 2a), or 60% of the 44-year mean haze days, which is a masking effect that must be overcome by any reduction in PM emissions in the next 44 years before the impact of the reduction can be statistically detected. Correspondingly, 44-year reductions required in YRD, PRD and SCB are 36, 67 and 24%, respectively (Supplementary Figs 7a, 8a and 9a, available as Supplementary Data at *NSR* online). These values are large but quite reasonable when the reductions are spread over 44 years. Figure 2b shows that the mean value of the BTH 10-year interannual variability in 2013–17 is about 16.7 haze days, or 62% of the mean haze days. Thus, a 62% or more reduction in PM emissions in 10 years is required to detect a statistically significant impact of the reduction. Similarly, 10-year reductions required for YRD, PRD and SCB are 34, 125 and 26%, respectively (Supplementary Figs 7b, 8b and 9b, available as Supplementary Data at *NSR* online). These 10-year reductions, except for PRD, are also reasonable for formulating effective control strategies. The emission reduction required for PRD is impractically large, reflecting the large variability of winter haze days in the 10-year period of 2007–16. Since no such large reduction of PM emissions occurred in PRD during 2007–16 (Supplementary Fig. 3, available as Supplementary Data at *NSR* online), again the observed decreasing trend in PRD in this period is likely caused, at least partially, by changes in meteorological and climate conditions.

The results above clearly show that, because of the masking effect posed by the interannual variability, relatively long-term (10 years or longer) emission control strategies are more realistic and preferred. In addition, meteorological and climate conditions need to be considered in formulating effective air pollution control strategies.

## SUMMARY OF MAJOR FINDINGS

Analyses of the time series of winter haze days in BTH reveal that there was a remarkable lack of trend in the winter haze days during a 40-year span from 1973 to 2012. This phenomenon is robust and widespread over the majority of provinces and special districts in China. The lack of trend is in a stark contradiction with PM emissions, which have increased by as much as 2.5-fold from 1973 to 2012. The contradiction strongly suggests that factors/processes other than PM emissions are in control of the trend and interannual variability of winter haze days. Our observation-based analysis suggests that meteorological and climate conditions are in control of the trend and interannual variability of winter haze days in the four regions.

Linear trends of winter haze days in BTH, YRD, PRD and SCB derived for the period 1973–2016 are 1.5%/year, 4.7%/year, 4.4%/year and no significant trend, respectively. In comparison, corresponding trends of PM emissions are 2.1%/year, 2.4%/year, 2.7%/year and 1.7%/year, respectively. The trends of haze days in the four regions show little resemblance with their corresponding trends of PM emissions, especially considering the large interannual variability of haze days. This again underscores the notion that climate and meteorological conditions rather than the PM emissions play the predominant role in controlling the interannual variability and thus the trends of haze days.

The major reason for the differences in trends among the four polluted regions is most likely due to a depression of the winter haze days during the period 1999–2012. Our analysis suggests that the depression is caused by a combination of relatively small values (relative to non-depression periods) of PDO, AO, ENSO and GT, in addition to relatively large values of EAWM and ASI. The depression apparently diminishes gradually toward southeastern China, as shown in YRD and PRD (detrended data in Fig. 1). This is the major cause of the differences in trends among the four polluted regions. Furthermore, in PRD, the part of the depression before 2005 has mostly diminished, while the part of the depression near 2008–12 remains robust and appears to have extended all the way to 2016.

Since the interannual variability is controlled by meteorological and climate conditions, which we lack the capability to predict, the interannual variability poses as a masking effect that must be overcome by any change in PM emissions before its impact becomes statistically detectable. We find that the interannual variability of the four regions ranges from 24 to 125%, depending on the regions and time intervals, most of which are so large that 10-year or longer control strategies are needed. Finally, meteorological and climate conditions need to be considered in formulating effective future air pollution control strategies.

## METHOD AND DATA

### Statistical analysis

Statistical significance tests are used extensively throughout this study. We apply the bootstrap method [30] to examine whether the key meteorological parameters in NDJF haze days are statistically different from the clean days. We first collect the daily NCEP/NCAR reanalysis [31] data and then regenerated the bootstrap samples with a size of *n* = 5000. The null hypothesis is that the haze days data and the clean days data are statistically from the same probability distribution with equal means. For those areas with *P*-values less than 0.01 (or 0.05), we reject the null hypothesis and conclude that the values in haze days over these areas are significantly different from the clean days at the 99% (or 95%) significant level.

### Study region

The area studied includes four major polluted regions of China (Supplementary Fig. 1, available as Supplementary Data at *NSR* online): BTH, YRD, PRD and Sichuan Basin (SCB). These regions are selected because they are four of the most polluted areas in China [32,33]. Only stations with mean winter haze days more than 3 days are chosen for the analysis. The numbers of stations chosen this way are 7, 16, 5 and 8 stations for the four regions, respectively.

### Data

Daily visibility is obtained from Global Summary of the Day (GSOD) database from National Environmental Satellite, Data, and Information Service (NESDIS) of the US Department of Commerce. The GSOD data undergo extensive automated quality control by the Air Weather Service, and over 400 algorithms are applied automatically to correctly ‘decode’ the synoptic data, and to eliminate many of the random and systematic errors found in the original data. Data are generally available from 1929 to the present. But data needed for this study are complete only after 1973.

Meteorological parameters are obtained from NCEP/NCAR reanalysis [31] and the boundary layer height is from ERA-Interim reanalysis [34]. GT is taken from the Global Historical Climatology Network-Monthly (GHCN-M) temperature dataset [35]. The Niño-3.4 index, an annual average of sea surface temperature (SST) over the domain of 5°S–5°N, 120°W–170°W in the tropical Pacific [36], available at http://origin.cpc.ncep.noaa.gov/products/analysis_monitoring/ensostuff/detrend.nino34.ascii.txt, was used as the index of ENSO. The AO index was collected from the Climate Prediction Center of the National Oceanic and Atmospheric Administration (NOAA) [37]. The PDO index was obtained from the University of Washington (from http://jisao.washington.edu/pdo/PDO.latest) [38]. The EAWM index was calculated following the approach proposed by Wang and Chen [39] with meteorological parameters obtained from NCEP/NCAR reanalysis. The ASI concentration data are derived from HadISST (Hadley Centre Sea Ice and Sea Surface Temperature data set) [36].

## Supplementary Material

nwy118_Supplemental_Files
