# Supplementary material for: An observation-based perspective of winter haze days in four major polluted regions of China
Source: Natl Sci Rev. 2018 Oct 17;6(3):515–23. doi: 10.1093/nsr/nwy118 (PMC8291446; doi:10.1093/nsr/nwy118)

**Supplementary Data**

**An observation-based perspective of winter haze days in four major polluted regions of China**

Lu Mao^1^, Run Liu^2^, Wenhui Liao^3^, Xuemei Wang^2^, Min Shao^1,2^, Shaw Chen Liu^2*^, Yuanhang Zhang^1*^

^1^State Key Joint Laboratory of Environmental Simulation and Pollution Control, Beijing Innovation Center for Engineering Science and Advanced Technology, College of Environmental Sciences and Engineering, Peking University, Beijing 100871, China

^2^Institute for Environmental and Climate Research, Jinan University, Guangzhou 510632, China

^3^Guangdong University of Finance, Guangzhou 510521, China

Corresponding Authors:

Shaw Chen Liu ([shawliu@jnu.edu.cn](mailto:shawliu@jnu.edu.cn)), Tel/Fax: +86-20-37336618.

Yuanhang Zhang ([yhzhang@pku.edu.cn](mailto:yhzhang@pku.edu.cn)), Tel/Fax: +86-10-62756592.

Table S1. The sample sizes of polluted days and clean days in four regions used to calculate Table 1.

| Region | Polluted days | Clean days |
| --- | --- | --- |
| BTH | 64 | 72 |
| YRD | 70 | 66 |
| PRD | 67 | 66 |
| SCB | 113 | 133 |

Figure S1. Four major polluted regions in China: Beijing-Tianjin-Hebei (BTH), Yangtze River Delta (YRD), Pearl River Delta (PRD) and Sichuan Basin (SCB). Solid circles represent stations with mean winter haze days more than 3 days, and open circles represent those less than 3 days.


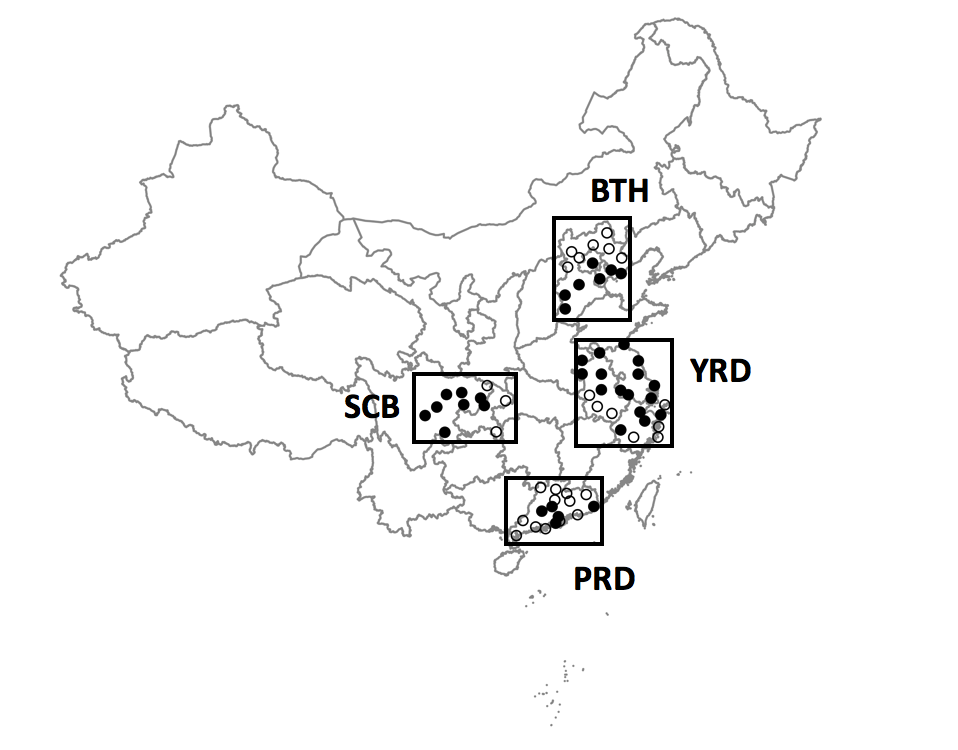


Figure S2. Distribution of monthly haze days in four major polluted regions in China. Error bars represent one-standard deviations of monthly haze days during the period 1973-2016. The percentage value in each figure indicates the yearly percentage of haze days occurred in the months of November, December, January, and February (NDJF).


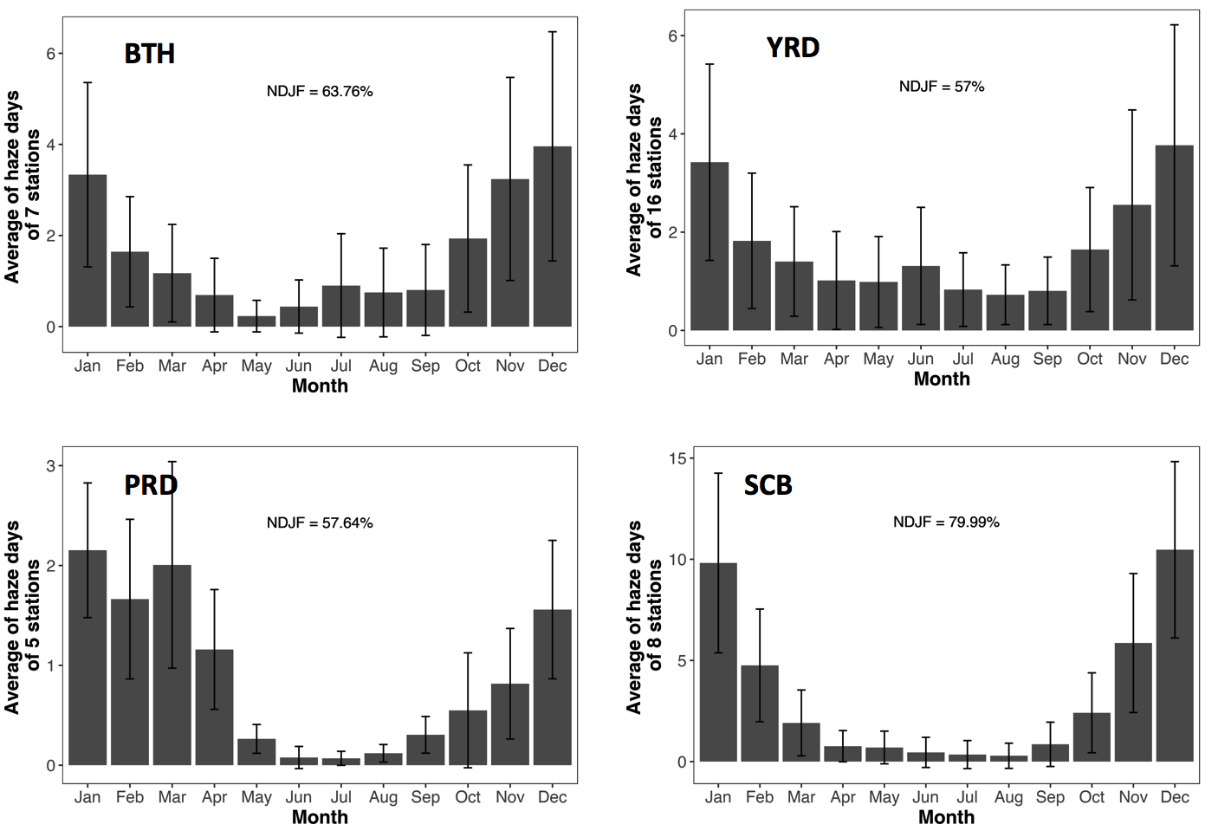


Figure S3. Temporal variations of emissions of major air pollutants in different regions. Dashed lines are linear regressions, and equations are statistics for the regressions. (Data source: PKU emission inventory, http://inventory.pku.edu.cn/)

Figure S4. Temporal variations of winter haze days in Heilongjiang, Liaoning, Shandong, Shanxi, Hubei, and Hunan provinces. Absolute values are shown in blue, detrended values in red. Dashed lines are linear regressions, and equations are statistics for the regressions.


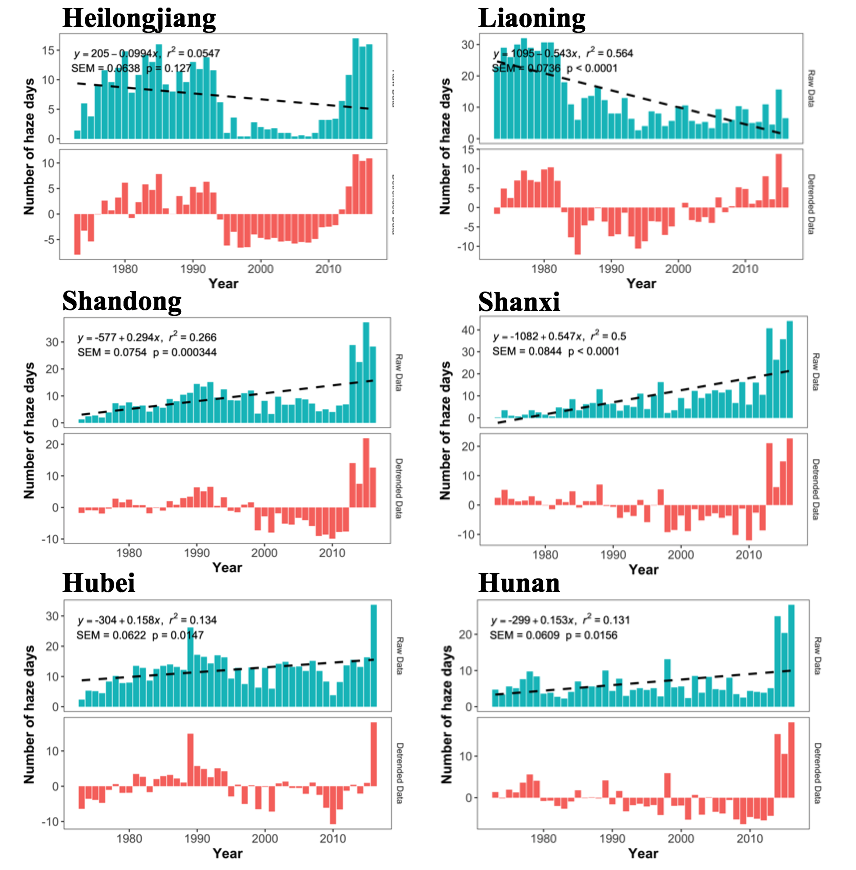


Figure S5. Temporal variations of winter haze days averaged over Jinzhou, Tsingtao, Shanghai and Hangzhou. Absolute values are shown in blue, detrended values in red. Dashed line is linear regression, and equations are statistics for the regression.


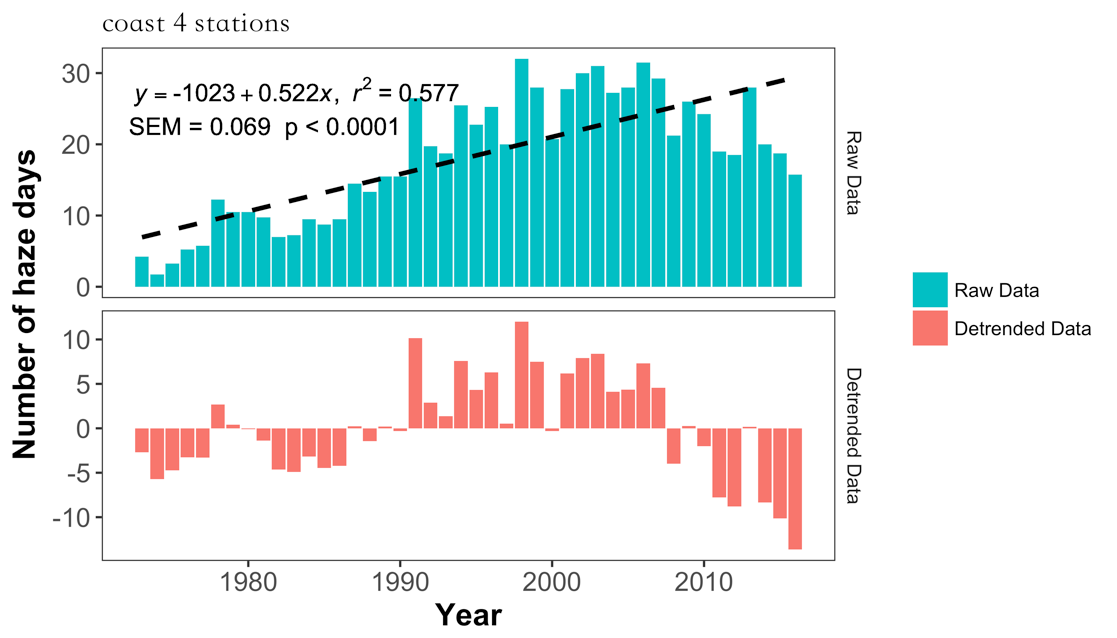


Figure S6. Trends in the boundary layer height, lapse rate and vertical velocity in BTH, YRD and PRD. Dashed lines are significant linear regressions, and equations are statistics for the regressions.

Figure S7. Interannual variability of winter haze days in Yangtze River Delta (YRD) in November-December-January-February for 44-year span (a), and ten-year span (b) during the period of 1973-2016. Solid bars denote mean values, shaded areas are 1-standard deviations.


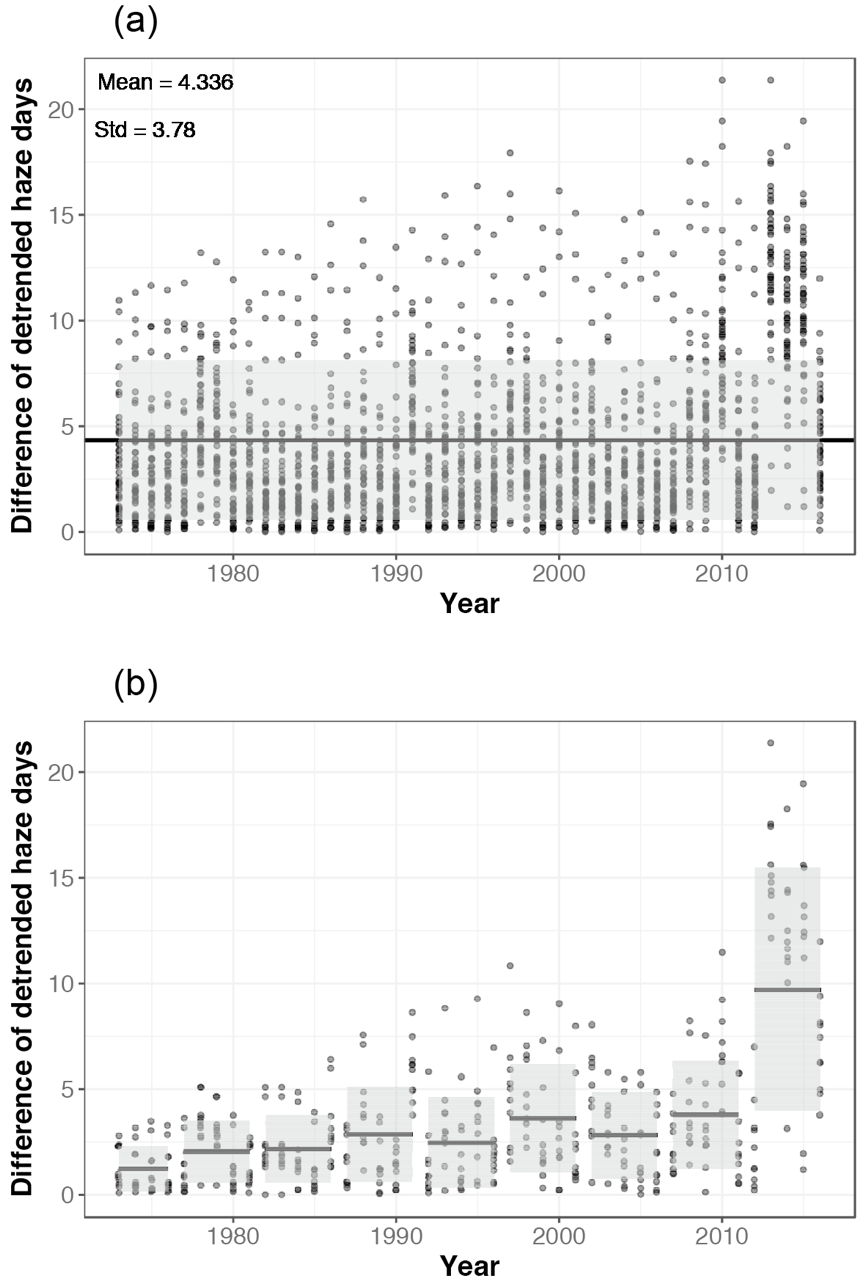


Figure S8. Interannual variability of winter haze days in Pearl River Delta (PRD) in November-December-January-February for 44-year span (a), and ten-year span (b) during the period of 1973-2016. Solid bars denote mean values, shaded areas are 1-standard deviations.


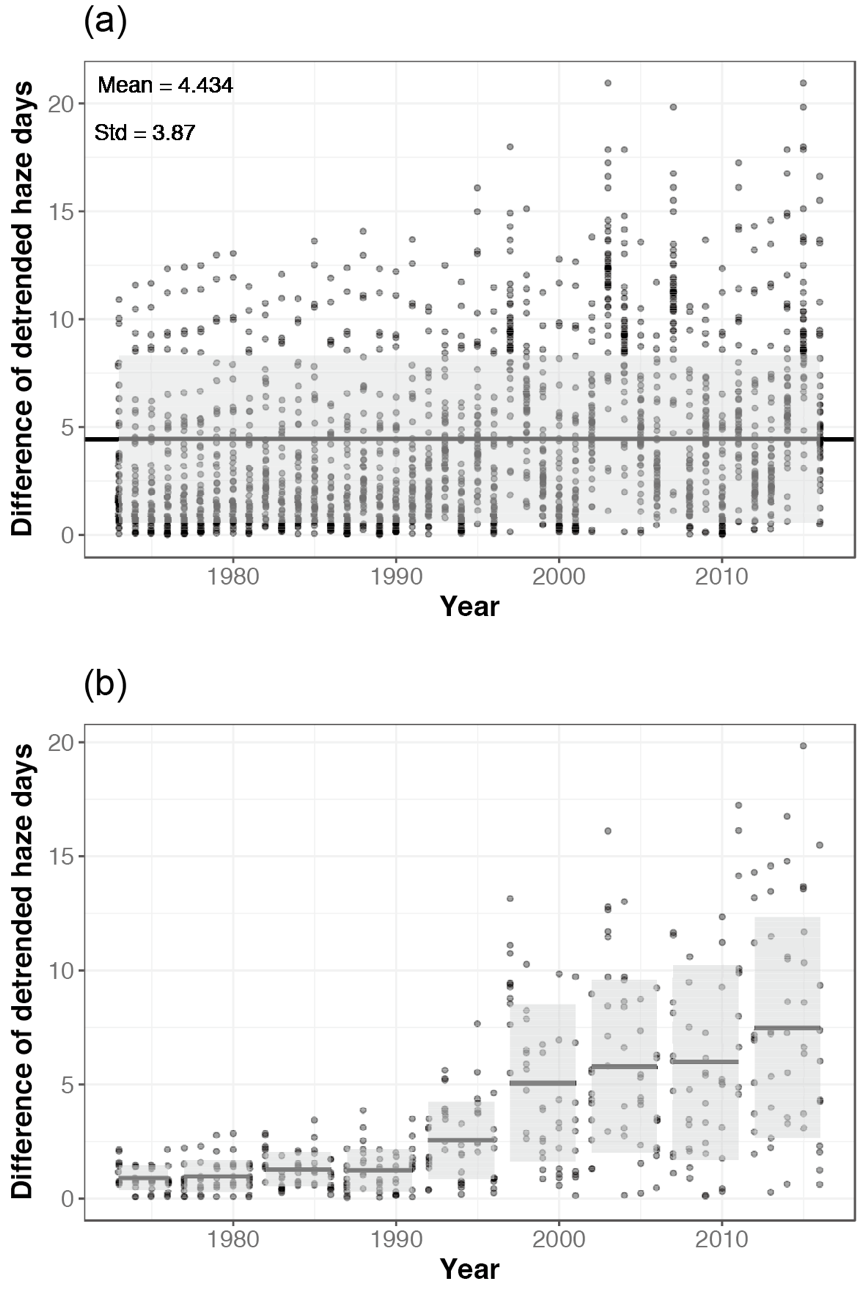


Figure S9. Interannual variability of winter haze days in Sichuan Basin (SCB) in November-December-January-February for 44-year span (a), and ten-year span (b) during the period of 1973-2016. Solid bars denote mean values, shaded areas are 1-standard deviations.


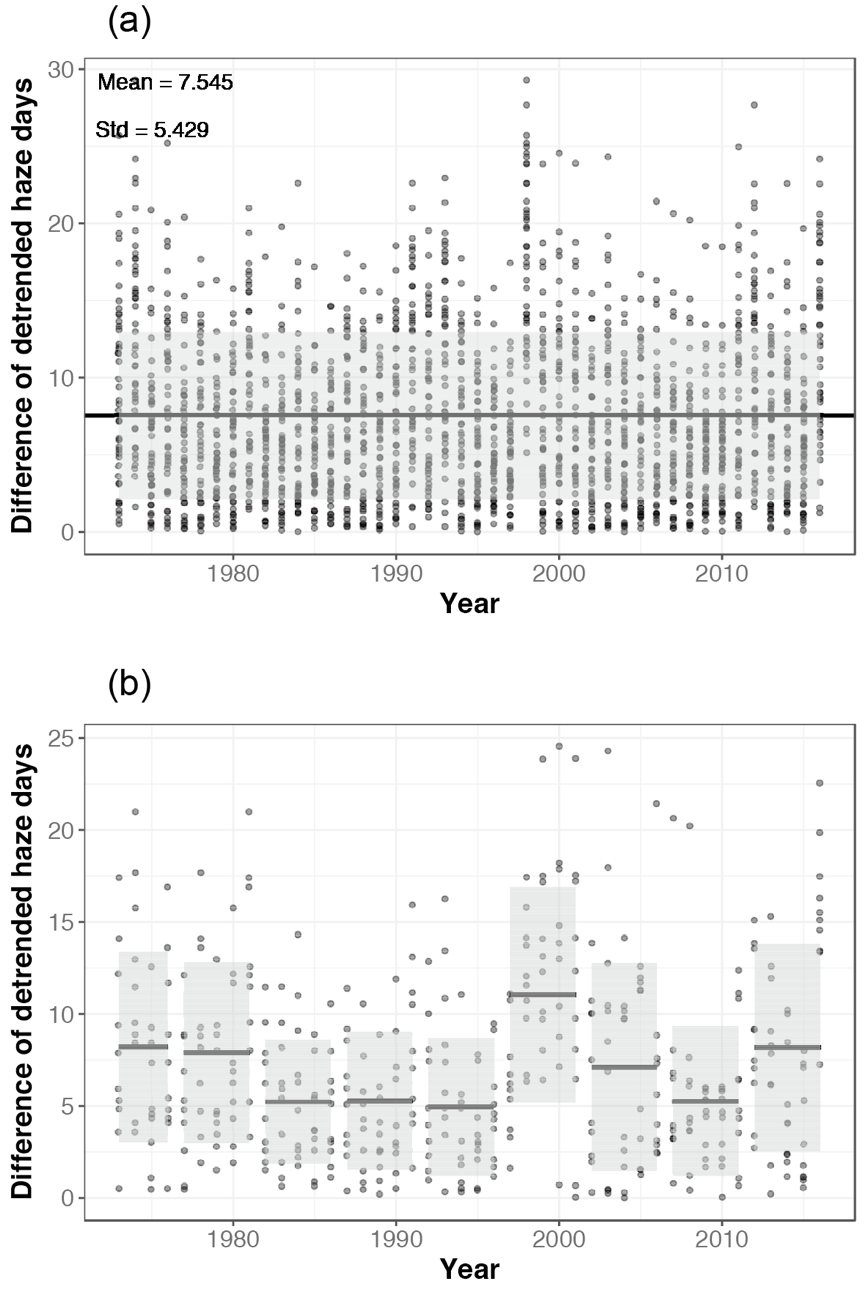

Supplement: nwy118_Supplemental_Files [file nwy118_supplemental_files.zip › SupplementaryData.docx]
