# Supplementary material for: An observation-based perspective of winter haze days in four major polluted regions of China
Source: Natl Sci Rev. 2018 Oct 17;6(3):515–23. doi: 10.1093/nsr/nwy118 (PMC8291446; doi:10.1093/nsr/nwy118)
Supplement: nwy118_Supplemental_Files [file nwy118_supplemental_files.zip › Response_Letter.docx]

Dear Editor,

We appreciate the prompt reviews and would like to thank the two reviewers for their valuable comments and suggestions on our revised manuscript entitled “An observation-based perspective of winter haze days in four major polluted regions of China” (No. NSR_MS-2018-019.R1). We have carefully considered all comments and suggestions. Listed below are our point-by-point responses to all comments and suggestions of the two reviewers (Reviewer’s points in black, our responses in blue).

**Reviewer #1**

I have read this MS carefully again. The issues raised in the early round have been satisfactorily addressed and there is no further question.

**Response:**

Thank you for your positive remarks.

**Reviewer #3**

The revision addressed some of my comments. A few comments (mine and other reviewers’) were not addressed well.

**Response:**

We highly value your comments and suggestions in this review as well as the review on our original manuscript. The two reviews are extremely thorough, perceptive and comprehensive. Our paper has benefited substantially from your reviews.

1.	What are the new approaches and findings of this work? Most of the analyses have been presented in previous studies.

**Response:**

You are correct that the approaches used in our study have been well established by previous works. Nevertheless, we believe that the three findings as quoted in the following from our abstract are new and significant: “*First, there was no significant trend in the number of winter haze days in most provinces and districts in eastern China from 1973 to 2012, contrary to the two-and-half-time increase in emissions of particulate matter and its precursors (PM emissions) in the same period of time. Second, meteorological and climate conditions rather than PM emissions are in control of the interannual variabilities and trends of winter haze days. These interannual variabilities (ranging from 24% to 125%) pose as a substantial masking effect that must be overcome by any control of PM emissions before its impact becoming statistically detectable*”. These three findings, to our limited knowledge, have not appeared in any previous publications. Finally, we would like to point out that the third finding uses a new approach – using observed historical interannual variabilities as masking effects for evaluating the effectiveness of historical as well as future emission control policies. Surely it is a relatively simple approach, yet it is new (to our limited knowledge), effective and useful.

In regard to the significance of our findings, we would like to respond by quoting reviewers #1 and #2 of the original manuscript as follows.

Original reviewer #1: “This is interesting and also is of great importance.”

Original reviewer #2: “the topic is timely, interesting and of potentially significance.”

In addition, quoting your own comment on our original manuscript: “some of the results look interesting”.

2.	The manuscript points out the differences in trend in four polluted regions but does not give insightful reasons for the differences.

**Response:**

This is a very good point, we appreciate it. We now realize that we have discussed “reasons for the differences in trends in four polluted regions” in various parts of the manuscript but failed to summarize them. For example, around lines 136–139 we state “*… the lack of trend in BTH and other regions can be attributed to a depression of the winter haze days during the period 1999–2012. The depression apparently diminishes gradually toward southeastern China as shown in YRD and PRD (Figure 1)*”; around lines 146–148 “*… in PRD the part of depression before 2005 has mostly diminished, while the part of depression near 2008–2012 remains robust. Furthermore, the 2008–2012 depression seems to have extended all the way to 2016 in PRD*”; and around lines167–170 “*As a brief summary for this paragraph, our analysis shows that the depression of winter haze days during 1999–2012 is caused by a combination of relative small (relative to non-depression periods) values of PDO, AO, ENSO and GT, in addition to relative large values of EAWM and ASI.*”

Thanks to your comment, we now add a summary of the “reasons for the differences” around line 252 in the second revision as shown below:

“*The major reason for the differences in trends among the four polluted regions is most likely due to a depression of the winter haze days during the period 1999–2012. Our analysis suggests that the depression is caused by a combination of relative small values (relative to non-depression periods) of PDO, AO, ENSO and GT, in addition to relative large values of EAWM and ASI. The depression apparently diminishes gradually toward southeastern China as shown in YRD and PRD (detrended data in Figure 1). This is the major cause of the differences in trends among the four polluted regions. Furthermore, in PRD the part of depression before 2005 has mostly diminished, while the part of depression near 2008–2012 remains robust and appears to have extended all the way to 2016.*”

We admit that even with this summary the reasons for the differences in trends in four polluted regions are still not insightful enough and need more definitive studies such as model simulations. We plan to do those simulations in our future studies.

3.	The explanation of the calculation of trend of emissions is not convincing. NMVOCs account for a large fraction of emissions but are expected to have small contributions to PM_2.5_ concentrations. The response says that 30% of PM_2.5_ is SOA, which needs to be supported by references. The yield of SOA from NMVOCs and it’s contribution to PM_2.5_ should not be presented by a quite casual way. The authors can justify by using results from previous studies.

**Response:**

We are sorry that our previous response was too simple and lacking in rigor, although we did include two references: “Huang et al., 2014; Sun et al., 2016”. We also apologize for simply and mistakenly saying that secondary organic aerosols (SOA) accounts for about 30% of PM_2.5_. We should have said that organic matter (OM, including SOA and primary organic matter) accounts for about 30% of PM_2.5_, and should have elaborated on this point. The elaboration is now respectively submitted in the following for your deliberation.

(1) Contributions of OM to PM_2.5_

The dominance of OM in PM_2.5_ has been widely observed at various sites in China. For instance, Huang et al. (2014) have found that OM (green portions of composition pie charts in Figure R1) accounts for 30%–50% of PM_2.5_ composition in typical haze events in Beijing, Shanghai, Guangzhou and Xi’an.

Figure R1. Chemical composition and source apportionment of PM_2.5_ collected during the high pollution events of 5–25 January 2013 at the urban sites of Beijing, Shanghai, Guangzhou and Xi’an (Figure 1 in Huang et al. 2014).

In another paper, Tao et al. (2017) reviewed and summarized studies published after 2000 on chemical composition of PM_2.5_ in different regions of eastern China on the seasonal basis. They found that the proportion of OM is highest in winter at most sites in BTH, ranging from about 40% to 50%, while this proportion is smaller in YRD and PRD, ranging from about 20% to 30% (Figure R2).

Figure R2. Seasonal PM_2.5_ and dominant chemical components in typical cities in BTH, YRD and PRD (Figures 4–6 in Tao et al. 2017).

(2) Contributions of SOA to PM_2.5_

Studies using aerosol mass spectrometer (AMS) and the positive matrix factorization technique have illustrated that oxygenated organic aerosols (OOA), which are considered as surrogates of SOA, could account for about 40% to 70% of organic aerosols (OA) in winter (Table R1).

Table R1. Comparison of the mass fractions of OOA to the total OA mass in some sites in China using Aerodyne AMS during wintertime.

| Sampling Site | Sampling Period | Season | Mass fraction of OOA to total OA | References |
| --- | --- | --- | --- | --- |
| Beijing | Jan. 1–Feb. 1, 2013 | Winter | 54% | Zhang et al. (2014) |
| Lanzhou | Jan. 10–Feb. 4, 2014 | Winter | 43% | Xu et al. (2016) |
| Ziyang | Dec, 2012–Jan, 2013 | Winter | 71% | Hu et al. (2016) |
| Nanjing | Jan. 4–Jan. 31, 2013 | Winter | 73% | Tang et al. (2014) |

Since OM is an important part of PM_2.5_ (ranging from 20% to 50% as mentioned above) and SOA is a major component of OM (40% to 70% from Table R1), it follows that the contributions of SOA to PM_2.5_ should be about 10% to 35%, consistent with the results of 12% to 26% in Huang et al. (2014) (pink portions of sources/factors pie charts in Figure R1).

Figure R3. Major sources of particulate matter in diﬀerent regions of China during 1980–2016. The y-axis in each box chart is the contribution (%) of each source type (Figure 2 in Zhu et al. 2018).

In a recent review of source apportionment studies from 1980 to 2016, Zhu et al. (2018) suggested that the average contribution of SOA to PM_2.5_ in four key air pollution regions in China (North, East, South, and Southwest China) ranges from 10% to 20% (Figure R3).

(3) Yields of SOA from NMVOCs

NMVOCs are crucial precursors for SOA, especially alkanes, alkenes, and aromatic hydrocarbons (Pankow et al., 2001; Chen et al., 2011). Their reactions with atmospheric oxidants (HO, NO_3_, and O_3_) generate secondary VOCs, such as semi-volatile and non-volatile organic compounds (Seinfeld et al., 2001; Dechapanya et al., 2003), and can then condense into the particle phase forming SOA (Seinfeld et al., 2001). Several studies have conducted chamber experiments (Pankow et al., 2001; Dechapanya et al., 2003; Chen et al., 2011) to estimate the yields of SOA from NMVOCs, which can be used in model predictions. However, the SOA yields generated by model predictions tend to underestimate the measured values (Ait-Helal et al., 2014; Fu et al., 2014) due to precursors not measured, such as some semi-volatile organic compounds (de Gouw et al., 2011).

In summary, OM is an important chemical composition of PM_2.5_, and SOA accounts for a major part of OM. However, significant questions and uncertainties remain in the photochemical mechanisms and conversion efficiencies from NMVOCs to SOA.

Finally, we would like to point out that in response to the Reviewer #2’s suggestions on the original manuscript, in the first revision we replaced the EDGAR emission inventory with PKU inventory (Figure R4, http://inventory.pku.edu.cn/) which is more consistent with Li et al. (2017) than EDGAR. Since NMVOCs have similar increasing trends as other air pollutants (Figure R5, taken from EDGAR emission inventory), the large increase in PM emissions between 1973 and 2014 remains essentially unchanged. This means that the trends of NMVOCs have little influence on the trends of PM emissions, and thus not mentioned in the manuscript.

Figure R4. Temporal variations of emissions of major air pollutants in four different regions in China in 1973–2014 from PKU emission inventory (http://inventory.pku.edu.cn/). Dashed lines are linear regressions, and equations are statistics for the regressions.

Figure R5. Temporal variations of emissions of NMVOCs in the four different regions. Dashed lines are linear regressions, and equations are statistics for the regressions (Data source: EDGAR v4.3).

**References**

Ait-Helal, W, Borbon, A, Sauvage, S, *et al.* Volatile and intermediate volatility organic compounds in suburban Paris: variability, origin and importance for SOA formation. *Atmospheric Chemistry and Physics*. 2014; **14**(19): 10439–64.

Chen, X, Hopke, PK, Carter, WPL. Secondary organic aerosol from ozonolysis of biogenic volatile organic compounds: chamber studies of particle and reactive oxygen species formation. *Environmental Science & Technology*. 2011; **45**(1): 276–82.

de Gouw, JA, Middlebrook, AM, Warneke, C, *et al.* Organic aerosol formation downwind from the deepwater horizon oil spill. *Science*. 2011; **331**(6022): 1295–9.

Dechapanya, W, Eusebi, A, Kimura, Y, *et al.* Secondary organic aerosol formation from aromatic precursors. 2. Mechanisms for lumped aromatic hydrocarbons. *Environmental Science & Technology*. 2003; **37**(16): 3671–9.

Fu, PQ, Kawamura, K, Chen, J, *et al.* Secondary production of organic aerosols from biogenic VOCs over Mt. Fuji, Japan. *Environmental Science & Technology*. 2014; **48**(15): 8491–7.

Hu, W, Hu, M, Hu, WW, *et al.* Characterization of submicron aerosols influenced by biomass burning at a site in the Sichuan Basin, southwestern China. *Atmospheric Chemistry and Physics*. 2016; **16**(20): 13213–30.

Huang, RJ, Zhang, YL, Bozzetti, C, *et al.* High secondary aerosol contribution to particulate pollution during haze events in China. *Nature*. 2014; **514**(7521): 218–22.

Li, M, Zhang, Q, Kurokawa, J, *et al.* MIX: a mosaic Asian anthropogenic emission inventory under the international collaboration framework of the MICS-Asia and HTAP. *Atmospheric Chemistry and Physics*. 2017; **17**(2): 935–63.

Pankow, JF, Seinfeld, JH, Asher, WE, *et al.* Modeling the formation of secondary organic aerosol. 1. Application of theoretical principles to measurements obtained in the α-pinene-, β-pinene-, sabinene-, Δ^3^-carene-, and cyclohexene-ozone systems. *Environmental Science & Technology*. 2001; **35**(6): 1164–72.

Seinfeld, JH, Erdakos, GB, Asher, WE, *et al.* Modeling the formation of secondary organic aerosol (SOA). 2. The predicted effects of relative humidity on aerosol formation in the α-pinene-, β-pinene-, sabinene-, Δ^3^-carene-, and cyclohexene-ozone systems. *Environmental Science & Technology*. 2001; **35**(15): 1806–17.

Sun, J, Wu, FK, Hu, B, *et al.* VOC characteristics, emissions and contributions to SOA formation during hazy episodes. *Atmospheric Environment*. 2016; **141**: 560–70.

Sun, YL, Wang, ZF, Du, W, *et al.* Long-term real-time measurements of aerosol particle composition in Beijing, China: seasonal variations, meteorological effects, and source analysis. *Atmospheric Chemistry and Physics*. 2015; **15**(17): 10149–65.

Tang, L, Zhang, Y, Sun, Y, *et al.* Components and optical properties of submicron aerosol during the lasting haze period in Nanjing. *Chinese Science Bulletin*. 2014; **59**(20): 1955–66.

Tao, J, Zhang, LM, Cao, JJ, *et al.* A review of current knowledge concerning PM_2.5_ chemical composition, aerosol optical properties and their relationships across China. *Atmospheric Chemistry and Physics*. 2017; **17**(15): 9485–518.

Xu, JZ, Shi, JS, Zhang, Q, *et al.* Wintertime organic and inorganic aerosols in Lanzhou, China: sources, processes, and comparison with the results during summer. *Atmospheric Chemistry and Physics*. 2016; **16**(23): 14937–57.

Zhang, JK, Sun, Y, Liu, ZR, *et al.* Characterization of submicron aerosols during a month of serious pollution in Beijing, 2013. *Atmospheric Chemistry and Physics*. 2014; **14**(6): 2887–903.

Zhu, YH, Huang, L, Li, JY, *et al.* Sources of particulate matter in China: Insights from source apportionment studies published in 1987–2017. *Environment International*. 2018; **115**: 343–57.
